# Coffin‐Lowry syndrome in a girl with 46,XX,t(X;11)(p22;p15)dn: Identification of *RPS6KA3* disruption by whole genome sequencing

**DOI:** 10.1002/ccr3.2826

**Published:** 2020-04-06

**Authors:** Kaori Yamoto, Hirotomo Saitsu, Yasuko Fujisawa, Fumiko Kato, Keiko Matsubara, Maki Fukami, Masayo Kagami, Tsutomu Ogata

**Affiliations:** ^1^ Department of Pediatrics Hamamatsu University School of Medicine Hamamatsu Japan; ^2^ Department of Biochemistry Hamamatsu University School of Medicine Hamamatsu Japan; ^3^ Department of Molecular Endocrinology National Research Institute for Child Health and Development Tokyo Japan

**Keywords:** Coffin‐Lowry syndrome, *RPS6KA3*, translocation, whole genome sequencing

## Abstract

We report a Japanese girl with Coffin‐Lowry syndrome phenotype such as hypertelorism, hypodontia, and tapering fingers and 46,XX,t(X;11)(p22;p15)dn. Whole genome sequencing revealed *RPS6KA3* disruption by the translocation, and X‐inactivation analysis indicated preferential inactivation of the normal X chromosome. The results explain the development of an X‐linked disease in this girl.

## Graphical abstract

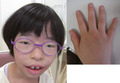

## 1 INTRODUCTION

Recent progress in molecular technology has permitted to identify the underlying causes in multiple genetic diseases. In particular, genome‐wide array comparative genomic hybridization (aCGH) and whole exome sequencing have successfully revealed various disease‐causing copy number variants and sequence variants.^1^, ^2^ Furthermore, whole genome sequencing (WGS), though it has not yet been performed widely in clinical practice, has been utilized as a powerful method to identify specific genes disrupted or impaired by chromosomal aberrations such as balanced translocations and inversions, because WGS can identify precise fusion points of such chromosomal aberrations.^3^, ^4^, ^5^ Here, we report *RPS6KA3* disruption identified by WGS in a girl with Coffin‐Lowry syndrome (CLS), an X‐linked disorder with phenotypic features usually being much severer in affected males than in affected females,^6^ and 46,XX,t(X;11)(p22;p15)dn.

## 2 CASE PRESENTATION

This Japanese girl was born to nonconsanguineous and healthy parents at 39 weeks of gestation after an uncomplicated pregnancy and delivery. At birth, her length was 48.0 cm (–0.4 SD), her weight was 2,480 g (–1.5 SD), and her occipitofrontal circumference (OFC) was 32.0 cm (–0.9 SD). She showed growth failure and developmental delay since birth. She controlled her head at ~5 months, walked without support at ~2.0 years of age, and spoke single words at ~2.5 years of age. Thus, she was placed on physical and occupational therapies from 3 years of age. She frequently had myoclonic seizures and received anticonvulsive drugs including sodium valproate in infancy to early childhood. Electroencephalograms and brain magnetic resonance imaging showed no discernible abnormalities. Seizures became infrequent with age and were not observed from 5 years of age.

**Figure 1:**
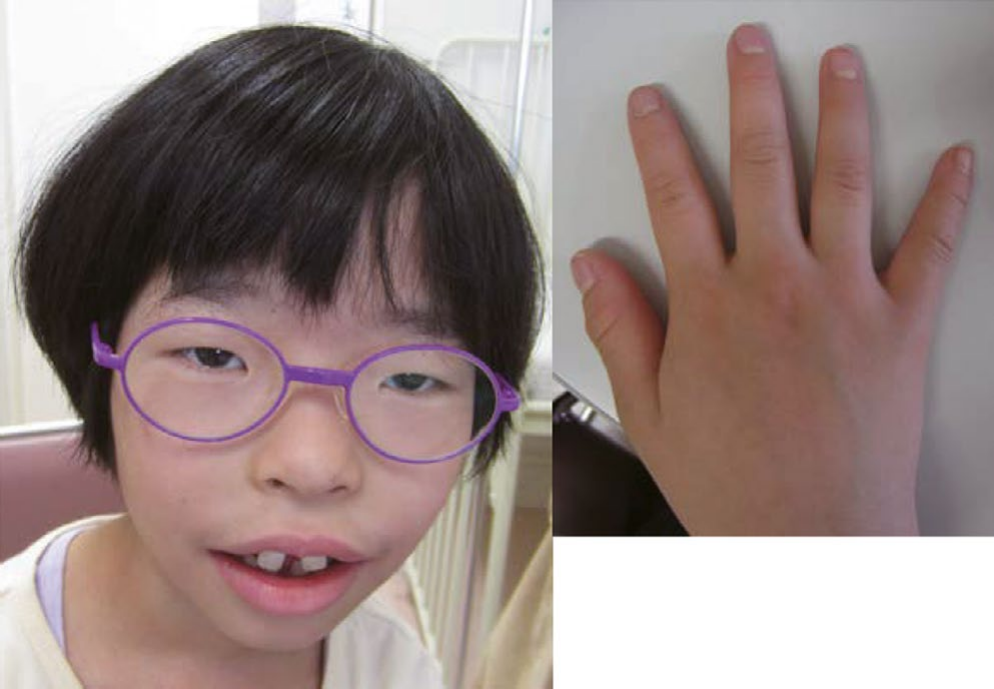
Photographs of this patient at six years of age

At 6 years of age, she was referred to Hamamatsu University Hospital because of short stature, multiple congenital anomalies, and developmental delay. Her length was 96.7 cm (–3.4 SD), her weight was 13.0 kg (–3.4 SD), and her OFC was 46.2 cm (–3.5 SD). Physical examination revealed CLS‐like features such as hypertelorism, myopia, broad nose with thick nasal alae and septum, maxillary hypoplasia, large open mouth with thick lips, hypodontia, widely spaced teeth, large medial incisors, and soft and fleshy hands with tapering fingers and short 5th fingers (Figure 1). Endocrine studies and bone survey for short stature were normal. Ophthalmologic and auditory examinations, and cardiovascular and visceral ultrasound studies, revealed no abnormal findings except for bilateral myopia (visual acuity, 0.4). Kyoto Scale of Psychological Development test^7^ indicated overall developmental quotient (DQ) of 26, cognitive‐adaptive DQ of 26, and language‐social DQ of 25; postural‐motor DQ was unmeasurable. Thus, it was suspected that she had CLS with a severe clinical presentation comparable to that of affected males or had some genetic abnormality leading to CLS‐like features.

Cytogenetic and molecular studies were performed for peripheral blood cells. This study was approved by the Institutional Review Board Committee at Hamamatsu University School of Medicine and was performed after obtaining written informed consent.

**Figure 2:**
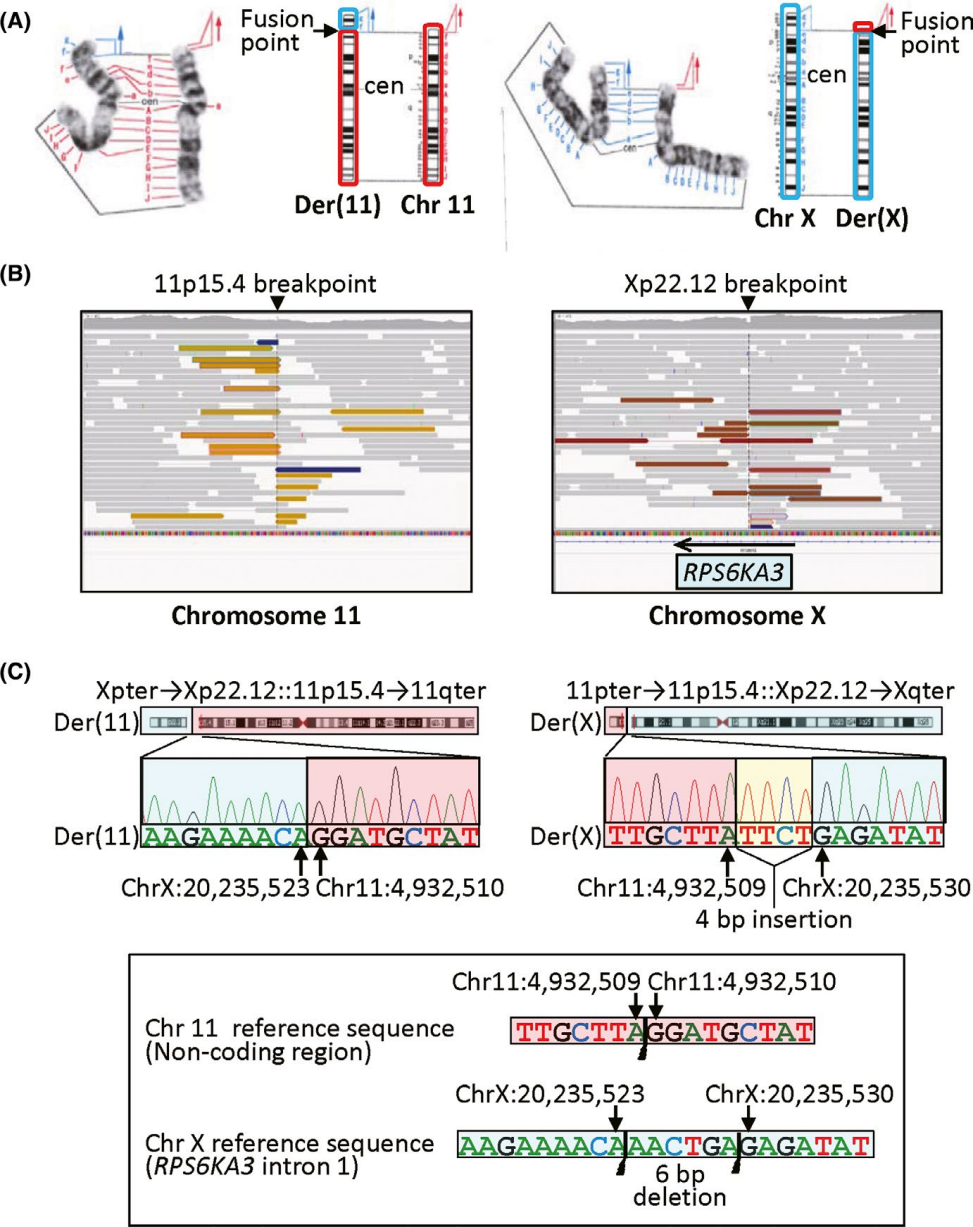
Summary of cytogenetic and molecular analyses. A, Partial G‐banding karyotype showing t(X;11)(p22;p15) and ideograms of normal and derivative chromosomes. B, Integrative Genomic Viewer (https://www.igv.org) screenshot of WGS pair‐end reads. C, The nucleotide sequences of the translocation fusion points on the der(11) and der(X) chromosomes. Sanger sequencing has been performed for PCR products obtained with the following primers: der(11), 5′‐TCCACTCTTTAGTGGGGAAAAA‐3′ and 5′‐GATGGTTGAGAGAGAGGCTGA‐3′; and der(X), 5′‐GCATTTTGAGACACCCTCCT‐3′ and 5′‐TGCCTCAAGAACACACTTCCT‐3′

Chromosome analysis revealed a de novo translocation, 46,XX,t(X;11)(p22;p15)dn, in all the 20 cells examined (Figure 2A). Subsequently, aCGH was carried out with a catalog human array (4 × 180K format, ID G4449A) (Agilent Technologies) according to the manufacturer′s instructions, revealing no deletion or duplication around the translocation fusion points (Figure S1), as well as no copy number variant absent from the parents or Database of Genomic Variants (http://dgv.tcag.ca/dgv/app/home) and ClinVar (http://www.ncbi.nlm.nih.gov/clinvar/).

Thus, we carried out WGS to identify the translocation fusion points, as reported previously.^4^ In brief, captured libraries were sequenced by HiSeq X Ten (Illumina) with 150 bp paired‐end reads, and structural variants were analyzed by Manta.^8^ Consequently, soft‐clipped, hard‐clipped, and discordant reads were found to be clustered at a noncoding region on 11p15.4 and at intron 1 of *RPS6KA3* on Xp22.12 (Figure 2B). Then, we performed direct sequencing for the PCR products obtained with primers flanking the translocation fusion points, revealing that the chromosomal fusion occurred between ChrX:20 235 523 (bp) and Chr11:4 932 510 (bp) in the der(11) chromosome and between Chr11:4 932 509 (bp) and ChrX:20 235 530 (bp) in association with an insertion of 4 bp segment of unknown origin in the der(X) chromosome; a 6 bp segment (ChrX:20 235 524‐20 235 529) was deleted from the X chromosome (according to GRCh37/hg19, https://genome.ucsc.edu) (Figure 2C). The break point of the chromosome 11 was located within long interspersed elements, and that of the X chromosome resided on a nonrepeat sequence (RepeatMasker, http://www.repeatmasker.org).

**Figure 3:**
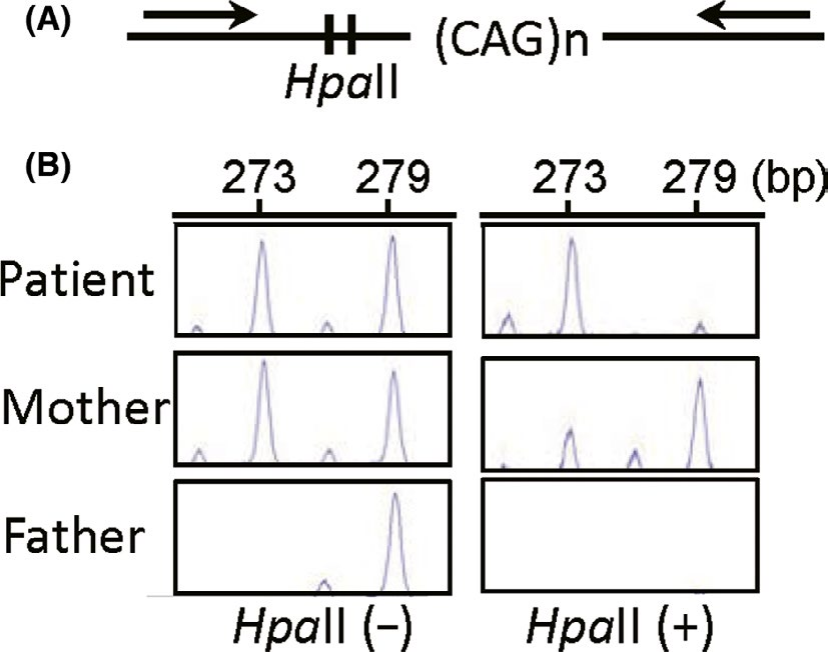
X‐inactivation analyses. A, The structure of *AR* exon 1 examined in this study. Arrows indicate the positions of a fluorescently labeled forward primer (5′‐TCCAGAATCTGTTCCAGAGCGTGC‐3′) and an unlabeled reverse primer (5′‐CTCTACGATGGGCTTGGGGAGAA‐3′). B, The PCR products visualized on the Applied Biosystems 3130 Genetic Analyzer using GeneScan (Life Technologies). The X‐inactivation ratio has been obtained as 87.3%:12.7% in the patient and 70.0%:30.0% in the mother

We further examined X‐inactivation pattern by analyzing the physical length and the area under curve of PCR products obtained with primers flanking the CAG repeat region and the two methylation‐sensitive *Hpa*II sites at exon 1 of *AR*, before and after *Hpa*II digestion (Figure 3A).^9^ The results showed a skewed, but not nonrandom, X‐inactivation pattern, with the inactivation ratio between the maternally derived X chromosome with a 273 bp allele and the paternally inherited X chromosome with a 279 bp allele being 87.3%:12.7% (Figure 3B). Considering the selective or preferential inactivation of the normal X chromosome in balanced X;autosome translocations,^10^ the results also indicated the presence of the 273 bp allele on the normal X chromosome and that of the 279 bp allele on the der(X) chromosome.

Lastly, we studied methylation patterns of the *H19*/*IGF2*:IG‐DMR and *KCNQ1OT1*:TSS‐DMR on chromosome 11p15.5,^11^ to examine possible spreading of X‐inactivation into the imprinted regions on the inactivated der(X) chromosome. Methylation‐specific multiplex ligation‐dependent probe amplification was carried out with the probe mixture kit ME030‐C3 BWS/RSS (MRC‐Holland, Amsterdam) before and after digestion with a methylation‐sensitive restriction enzyme *Hha*I, revealing obviously normal genome copy number for *Hha*I‐undigested DNA and apparently normal methylation pattern for *Hha*I‐digested DNA (Figure S2).

## 3 DISCUSSION

WGS successfully identified the fusion points of the X;11 translocation, thereby revealing the disruption of *RPS6KA3* in this girl and determining her precise karyotype as 46,XX,t(X;11)(p22.12;p15.4)dn. This provides further support for the usefulness of WGS in the determination of the precise fusion points in chromosomal aberrations.^3^, ^4^, ^5^ It should be pointed out, however, that WGS with short paired‐end reads (~150 bp long) utilized in this study is not always able to identify chromosomal fusion points.^3^, ^4^ WGS with long paired‐end reads (several kb long) may be capable of revealing complicated fusion points generated by several mechanisms such as chromothripsis, chromoanasynthesis, and chromoplexy.^12^

The X‐inactivation analysis implies functional silencing of *RPS6KA3* in most cells with an inactive normal X chromosome, and functional disomy for the translocated Xpter–p22.12 region and possible functional monosomy for the translocated 11pter–p15.4 region in a small portion of cells with an inactive der(X) chromosome. The results would explain why this girl exhibited typical CLS phenotype as observed in affected males, as well as severe short stature and developmental delay. To our knowledge, this is the first report describing a female with typical CLS because of a balanced X;autosome translocation disrupting *RPS6KA3*.

Several matters are also notable in this study. First, the translocation fusion points were associated with a 4 bp insertion and a 6 bp deletion. This implies that the translocation was mediated by nonhomologous end joining (NHEJ).^13^ Indeed, NHEJ is the predominant mechanism for the development of nonrecurrent balanced translocations.^5^ Second, X‐inactivation analysis showed a preferential, but not nonrandom, X‐inactivation pattern. This would be due to the smallness of the translocated regions, because the cell selection effect of functional disomy for the Xpter–p22.12 region and possible functional monosomy for the 11pter–p15.4 region would remain mild. Indeed, in balanced X;autosome translocations, variable degrees of random X‐inactivation are predominantly found in patients with X chromosomal break points at Xp22 or Xq28.^14^ Third, the methylation patterns of the *H19*/*IGF2*:IG‐DMR and *KCNQ1OT1*:TSS‐DMR were apparently normal. Although the frequency of the inactivated der(X) chromosome remain minor, this would argue against the spreading of X‐inactivation into the *KCNQ1OT1*:TSS‐DMR that is normally unmethylated on the paternally inherited chromosome 11 (the data of the *H19*/*IGF2*:IG‐DMR are not informative, because this DMR is normally methylated when paternally inherited).^11^ Indeed, to keep the differentially methylated pattern, the imprinted regions may be structurally isolated,^15^ and such a structural property may have protected from the spreading of the X‐inactivation. It is unknown, however, whether the X‐inactivation has spread into nonimprinted regions on the translocated 11pter–p15.4 region.

In summary, we identified translocation break points disrupting *RPS6KA3* using WGS data in a female with CLS. This study argues for the value of WGS in the determination of the precise fusion points in balanced translocations.

## 4 CONCLUSION

We identified *RPS6KA3* disruption by WGS in a Japanese girl with CLS phenotype and 46,XX,t(X;11)(p22;p15)dn accompanied by preferential inactivation of the normal X chromosome. The results provide further support for the value of WGS in the determination of the precise fusion points in chromosomal aberrations.

## CONFLICT OF INTEREST

None declared.

## AUTHOR CONTRIBUTIONS

KY, HS, FK, KM, MF, and MK performed molecular studies. YF and TO performed the patient care and collected clinical information and blood samples. TO coordinated this study and wrote the paper.

## Supporting information

Figure S1

Figure S2
