# Supplementary figures and images for: Coffin‐Lowry syndrome in a girl with 46,XX,t(X;11)(p22;p15)dn: Identification of RPS6KA3 disruption by whole genome sequencing
Source: Clin Case Rep. 2020 Apr 6;8(6):1076–80. doi: 10.1002/ccr3.2826 (PMC7303873; doi:10.1002/ccr3.2826)

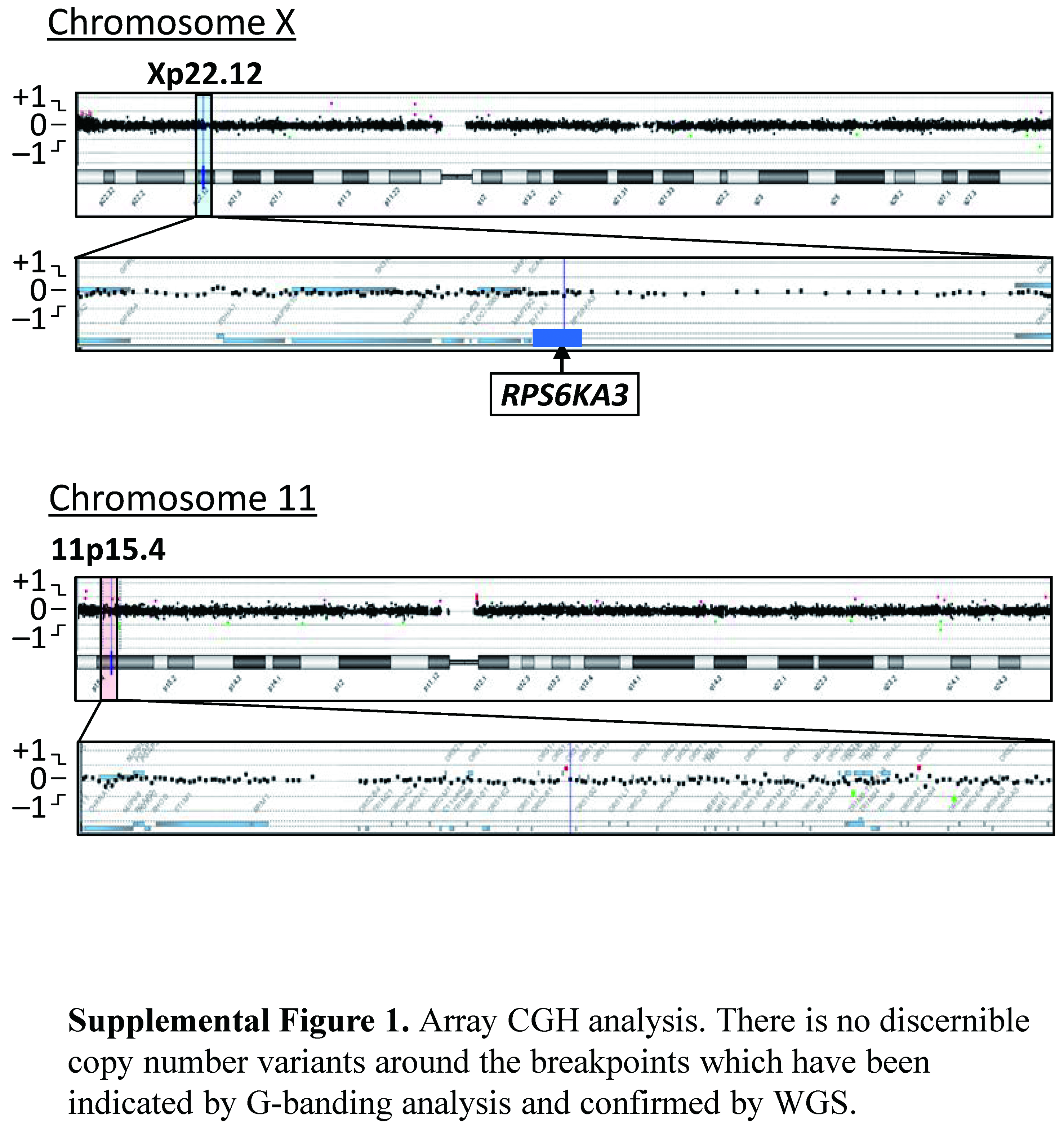

Supplement: Supplementary file 1 — Figure S1 [file CCR3-8-1076-s001.tiff]

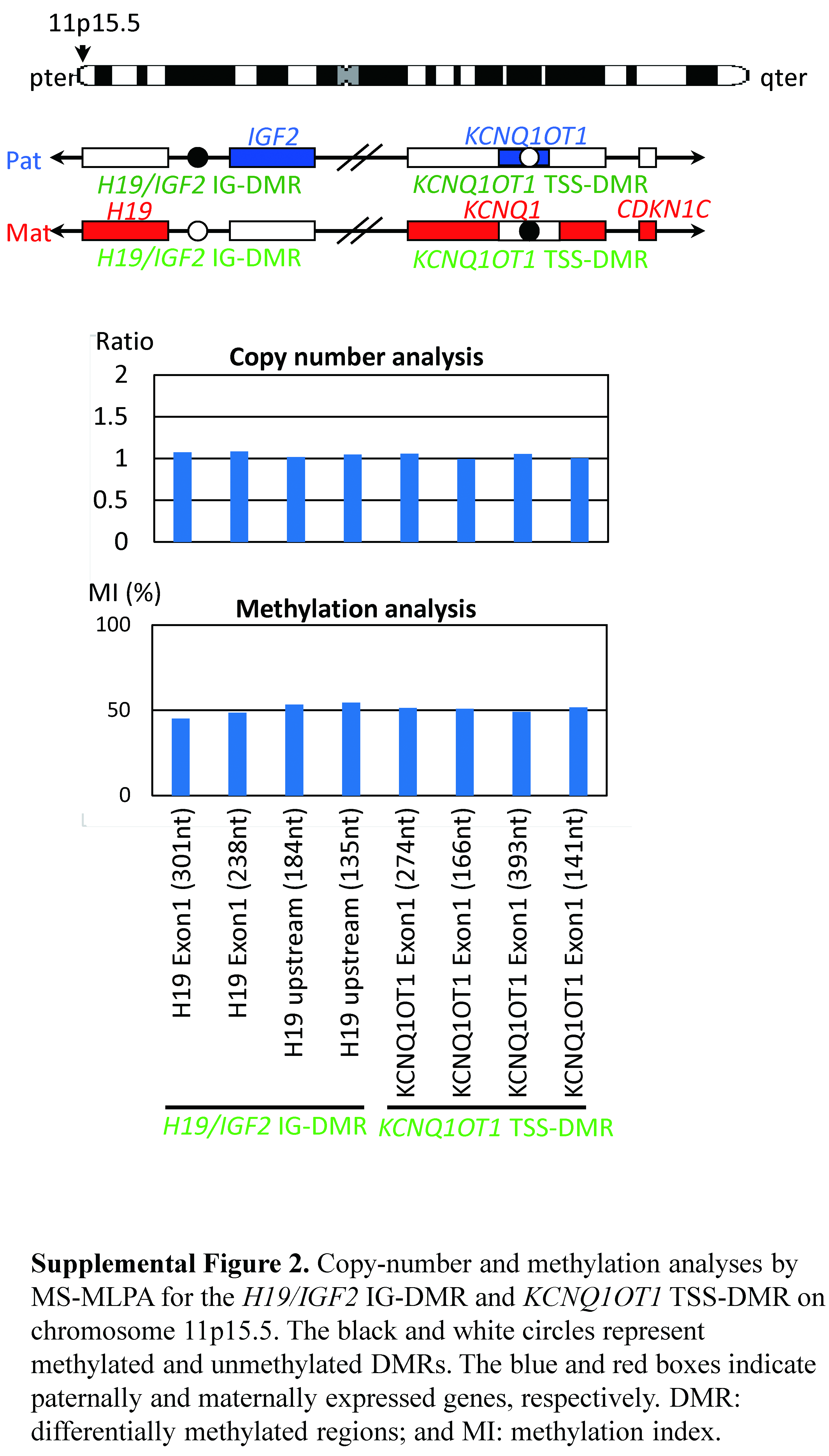

Supplement: Supplementary file 2 — Figure S2 [file CCR3-8-1076-s002.tiff]
